# GNSS Constraints to Active Tectonic Deformations of the South American Continental Margin in Ecuador

**DOI:** 10.3390/s21124003

**Published:** 2021-06-10

**Authors:** José Tamay, Jesús Galindo-Zaldivar, John Soto, Antonio J. Gil

**Affiliations:** 1 Departamento de Geociencias, Universidad Técnica Particular de Loja, San Cayetano Alto, Apartado Postal, Loja 11-01-608, Ecuador; jvtamay@utpl.edu.ec (J.T.); jesoto@utpl.edu.ec (J.S.); 2 Departamento de Geodinámica, Facultad de Ciencias, Universidad de Granada, 18071 Granada, Spain; 3 Instituto Andaluz de Ciencias de la Tierra (IACT)—CSIC, Universidad de Granada, 18071 Granada, Spain; 4 Departamento de Ingeniería Cartográfica, Geodésica y Fotogrametría, Campus de las Lagunillas, Universidad de Jaén, 23071 Jaén, Spain; ajgil@ujaen.es; 5 Centro de Estudios Avanzados en Ciencias de la Tierra, Energía y Medio Ambiente (CEACTEMA), Campus de las Lagunillas, Universidad de Jaén, 23071 Jaén, Spain

**Keywords:** GPS networks, active tectonics, transfer faults, Ecuadorian Andes, fore-arc basin

## Abstract

GNSS observations constitute the main tool to reveal Earth’s crustal deformations in order to improve the identification of geological hazards. The Ecuadorian Andes were formed by Nazca Plate subduction below the Pacific margin of the South American Plate. Active tectonic-related deformation continues to present, and it is constrained by 135 GPS stations of the RENAGE and REGME deployed by the IGM in Ecuador (1995.4–2011.0). They show a regional ENE displacement, increasing towards the N, of the deformed North Andean Sliver in respect to the South American Plate and Inca Sliver relatively stable areas. The heterogeneous displacements towards the NNE of the North Andean Sliver are interpreted as consequences of the coupling of the Carnegie Ridge in the subduction zone. The Dolores–Guayaquil megashear constitutes its southeastern boundary and includes the dextral to normal transfer Pallatanga fault, that develops the Guayaquil Gulf. This fault extends northeastward along the central part of the Cordillera Real, in relay with the reverse dextral Cosanga–Chingual fault and finally followed by the reverse dextral Sub-Andean fault zone. While the Ecuadorian margin and Andes is affected by ENE–WSW shortening, the easternmost Manabí Basin located in between the Cordillera Costanera and the Cordillera Occidental of the Andes, underwent moderate ENE–WSW extension and constitutes an active fore-arc basin of the Nazca plate subduction. The integration of the GPS and seismic data evidences that highest rates of deformation and the highest tectonic hazards in Ecuador are linked: to the subduction zone located in the coastal area; to the Pallatanga transfer fault; and to the Eastern Andes Sub-Andean faults.

## 1. Introduction

The subduction of oceanic plates at convergent tectonic boundaries is responsible for the release of more than 90% of the total seismic moment, mainly occurring along the subduction interface [1,2] and the development of first-order large elongated cordilleras [3]. Scientific research has shown that the subduction zone along the northwestern edge of America is one of the most active convergent margins in the world [4,5,6,7,8,9,10,11]. 

**Figure 1:**
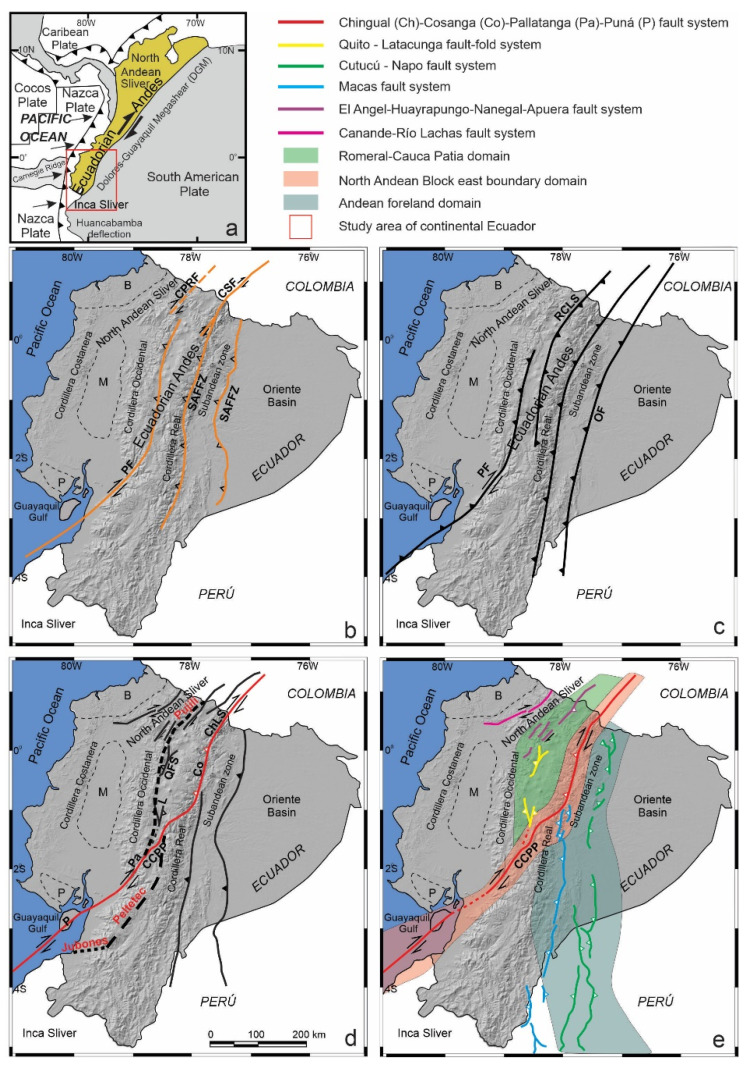
Main tectonic domains and structures proposed for the South American Plate margin in Ecuador. (**a**) Location of the North Andean Domain. (**b**) Proposal by [17]; Cauca Patia and Romeral Fault, CPRF; Chingual–La Sofía fault, CSF; Sub-Andean Front Fault Zone, SAFFZ. (**c**) Proposal by [18]; Pallatanga Fault, PF; Río Chingual La Sofía, RCLS; Oriente Fault, OF. (**d**) Proposal by [16]; Chingual–Cosanga–Pallatanga–Puná Fault Zone, CCPP (Chingual–La Sofía fault, ChL; Cosanga fault, Co; Pallatanga fault, Pa; Puná fault, P); Quito Fault, Q; Latacunga Fault, L. (**e**) Proposal by [2]; Define different domains according to the description given for oblique convergence tectonics. Borbón Basin, B; Manabí Basin, M; Progreso Basin, P.

**Figure 2:**
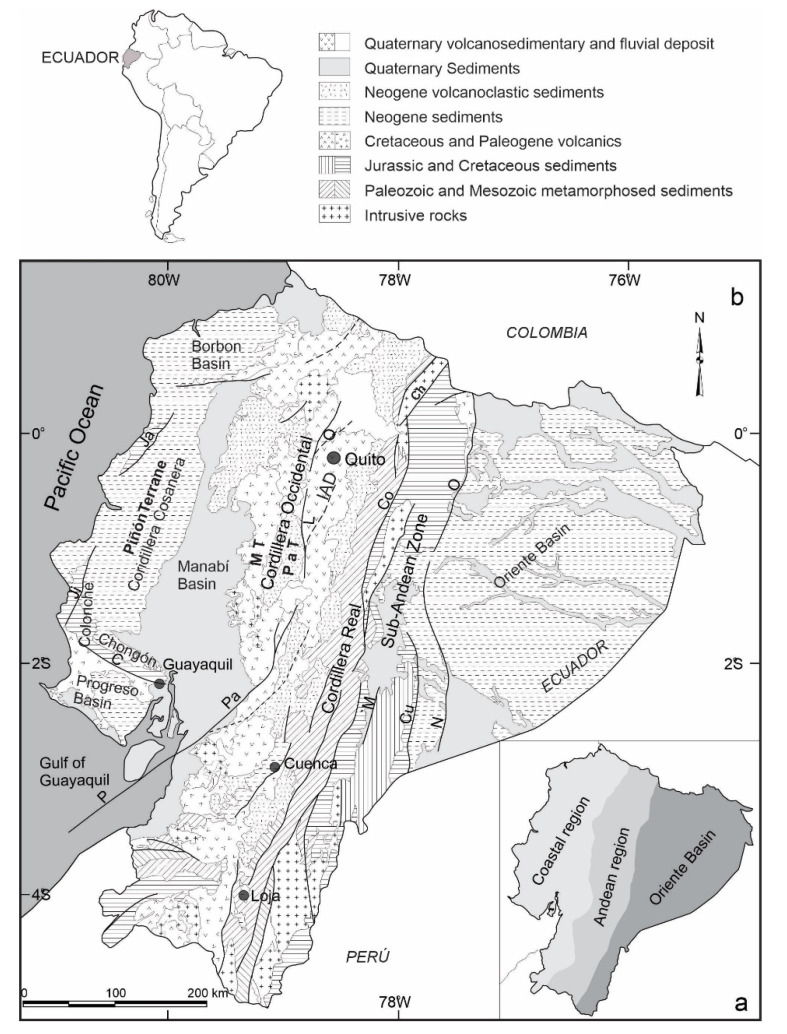
Subdivision regions of Ecuador (**a**). Simplified geological map of Ecuador, after [30] (**b**). Inter-Andean Depression, IAD; Puná Fault, P; Pallatanga Fault, Pa; Pallatanga Terrain, PaT; Pujilí Fault, Pu; Chingual–La Sofia Fault, ChL; Cosanga Fault, Co; Salado Fault, S; Macuchi Terrane, MT.

Oblique convergence leads to strain partitioning in thrust and shear components that are, respectively, perpendicular and parallel to the trench [12] and may give rise to the development of large continental domains bounded by faults. Moreover, convergent continental boundaries may undergo extension adjacent to shortening in fore-arc basins, as seen in Baja California [13,14]. These processes occur in the Ecuadorian Andes (Figure 1 and Figure 2), where the North Andean Sliver [15,16] is affected by shortening and extension and displaced northeastward from the South American Plate, favored by the Chingual–Cosanga–Pallatanga–Puná fault system [16] (Figure 1e). In this framework, discussion of the structures that accommodate deformation involving the main active faults and domains of the South American Plate margin in Ecuador has led to different proposals [2,16,17,18] (Figure 1). 

Geodetic GNSS (Global Navigation Satellite System) measurements are widely used for precise monitoring of the velocity vectors in active tectonic areas where seismicity is also located. The pattern of surface strain reflects the behavior of plate boundaries. Thus, the relative motion of the Nazca, Cocos, Caribbean and South America Plates constrains shortening along the northwestern South America Plate boundary [19,20,21,22,23,24,25]. 

The available GPS data in Ecuador from RENAGE and REGME databases [26,27] support regional displacements with respect to the South American Plate, showing in detail the heterogeneous tectonic behavior of each region. Most proposals support northeastward displacement of the North Andean Sliver, considered a main crustal block, as well as the activity of the Chingual–Cosanga–Pallatanga–Puná fault system [16]. Along the Inter-Andean Depression this system would form a restraining bend along the central and northern Ecuadorian Andes [17,28]. 

**Figure 3:**
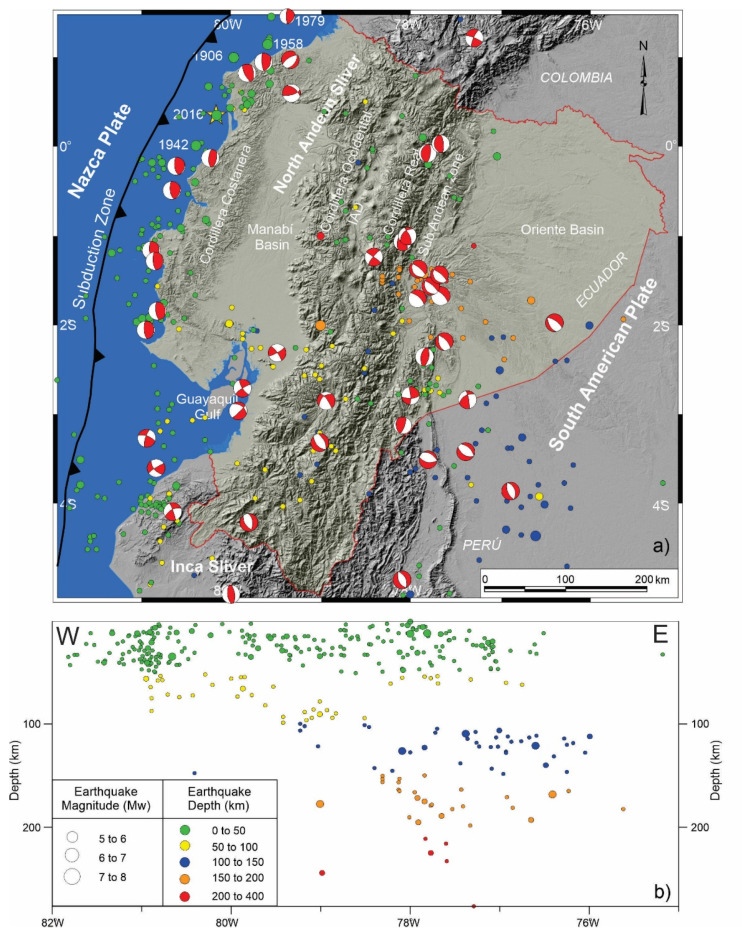
Seismicity distribution in Ecuador. The focal mechanisms associate the possible rupture zone. (**a**) Epicenters from the unified earthquake catalog 1976–2009. The main catastrophic historical earthquakes are indicated. (**b**) Depth distribution of earthquakes with >5 Mw. Data from Instituto Geofísico of Ecuador, after [49].

Along the western South American coastline, including Ecuador (Figure 3), most earthquakes are related to subduction of the Nazca Plate under the South American Plate (Figure 1a) [8,15,27] including shallow crustal seismicity in the Andes Cordillera and shallow to intermediate seismicity associated with the subducting slab. Since the early twentieth century, Ecuador has been damaged by several major earthquakes. The largest one occurred in 1906, with a magnitude of 8.8 (Mw) and a 500–600 km long break along the coast of Ecuador and Colombia, that caused a large tsunami. It was followed by earthquakes in the same area in 1942 (Mw = 7.8), 1958 (Mw = 7.7), 1979 (Mw = 8.2) and 2016 (Mw = 7.9) [4,6,8,10,11]. Such a concentration of earthquakes suggests the presence of asperities in the plate rupture [8,11,15,27] with especial relevance focused on the subduction of the Carnegie Ridge [8,11,29]. 

The aim of this research is to analyze and discuss the kinematics of the main tectonic features in Ecuador, crossing from the coast, the Ecuadorian Andes up to the Oriente foreland basin, based on the available GPS displacement and seismicity data. The mapping of areas with homogeneous active tectonic behavior allows us to improve our knowledge of the active tectonic deformations patterns and will contribute to improving the seismic hazard studies. 

## 2. Geological Setting

### 2.1. The Ecuador Continental Margin

Ecuador is located in northwestern South America, between latitudes 1° N and 4° S, crossed by the NNE-SSW Andes Cordillera (Figure 1), formed by the repeated accretion of allochthonous terranes against the northwestern margin of the South American Plate. Ecuador harbors three predominant structural domains: the coastal region (fore-arc); the Andean region (volcanic arc) and the Oriente Basin (foreland) (Figure 2a). 

The coastal region comprises the NNE–SSW and NW–SE branches of Cordillera Costanera and Chongón–Colonche. In addition, this region includes the Progreso, Manabí and Borbón basins [31,32] (Figure 2b). Allochthonous oceanic terrains were accreted to the continental margin during the Cretaceous [33,34]. Volcano-clastic and turbiditic sequences of latest Cretaceous and Paleogene age were derived from the Macuchi Terrain. Shallow marine sediments were deposited during Oligocene–Miocene, followed by Miocene to Quaternary sub-areal flood plains and alluvial deposits that are coeval with volcanic activity [34,35,36]. 

The Andean region (Figure 2) consists of the NNE–SSW Real and Occidental Cordilleras with active volcanoes separated by the Inter-Andean Depression. The Cordillera Real is mostly composed by sub-linear belts of Palaeozoic to Mesozoic metamorphic rocks intruded by S-type and I-type granite bodies [37,38,39]. The Cordillera Occidental is formed by two accreted oceanic terranes: the Macuchi terrane to the west and the Pallatanga terrane to the east. The Pallatanga terrane consists of early to late Cretaceous oceanic plateau rocks, a probable late Cretaceous tectonic melange and marine turbidites. The Macuchi terrane consists of an early Eocene, basaltic to andesitic, volcano sedimentary island arc sequence [40,41,42] (Figure 2). The Interandean Depression is filled by late Miocene–Holocene alluvial fans, fluvial and lacustrine facies, and volcanic deposits up to 1400 m thick [28] controlled by a fault system [17,43,44,45]. Eastward, the Sub-Andean zone is formed by Jurassic to Oligocene–Early Miocene metasedimentary rocks, overlying a Jurassic granitic batholith [37,39].

The Oriente Basin is formed by the Precambrian Guyana shield basement overlain by Paleozoic marine sediments, Triassic–Jurassic marine and continental rift deposits and Late Jurassic volcanoclastic sediments [46]. These, in turn, are unconformably overlain by a shallow marine to continental Cretaceous sedimentary series [46,47,48], and Paleogene and Neogene to Recent continental and shallow marine sediments [47].

### 2.2. Recent Tectonic Structures

The tectonics of Ecuador is influenced by three main tectonic elements: The Nazca and South America plates and the North Andean Sliver located in between (Figure 1a). Moreover, seismicity and volcanism show a high degree of segmentation along the strike of the Andes Cordillera due to the subduction of Carnegie Ridge [8,29]. 

The geodynamics of the North Andean Sliver is controlled through the Pallatanga Fault [40]. This fault system extends east of the Gulf of Guayaquil [43] up to the Inter-Andean Depression. There are different proposals regarding the detailed structure of the fault system. The authors of [17] hold that Cauca Patia and Romeral faults reach the margin of the Cordillera Occidental, and that the Pallatanga fault is located along the Inter-Andean Depression. The Chingual–La Sofia fault furthermore affects the Cordillera Real and Sub Andean front (Figure 1b). The authors of [18] confirm the presence of the Pallatanga fault, but modify the trace of the Chingual–La Sofia Fault, extending it toward the western border of the Cordillera Real, forming a boundary with the Inter-Andean Depression (Figure 1c). The authors of [16] propose the continuity of the Chingual–Cosanga–Pallatanga–Puná fault zone, which represents the best-developed fault zone in Ecuador, over 800 km long (Figure 1d). The Inter-Andean Depression is deformed by Quito and Latacunga N–S-oriented fault zones, curved westward up to NNE–SSW and apparently rooted in the Pujilí Suture zone (Figure 1d). A new geodynamic model for oblique convergence tectonics of Ecuador [2], considers three main fault segments: (a) the Romeral-Cauca–Patia domain (formed by the Quito and Latacunga fault zone in Ecuador and El Angel fault system in Colombia), (b) the North Andean fault zone that encompasses strike-slip and reverse faults (includes four segments: the Chingual–Cosanga–Pallatanga and Puná fault) and (c) the Andean foreland, subdivided in Napo, Cutucú and the Moyobamba fault zones at the boundary with the South American Plate (Figure 1e). 

### 2.3. Seismicity

The region has undergone numerous earthquakes in historical and instrumental periods [49] (Figure 3). Local seismic station coverage is dense enough to allow for determination of hypocentral depths [49,50] and earthquake focal mechanisms [50,51]. Regional seismicity shows a heterogeneous distribution, including shallow and intermediate seismicity reaching roughly 200 km depth related to the subduction of the Nazca Plate below the North Andean Sliver and South American Plate (Figure 3). In Andes Cordillera, the shallow seismicity extends from Cordillera Occidental up to the Sub-Andean zone. Southward in the Ecuadorian Andes, the seismicity is distributed over a broad area including intermediate seismicity in the foreland Oriente Basin. Another seismicity band is located parallel to the coast close to the subduction trench. 

The continental margin in Ecuador undergoing active subduction has a seismic segmentation most likely caused by deep transverse faults [8]. Five main catastrophic historical earthquakes occurred in this region: in 1906, with Mw = 8.8 and a breaking length of 500 km [4,52]; in 1942 (Mw = 7.8); in 1958 (Mw = 7.7); in 1979 (Mw = 8.2) [4,8,10] and 2016 (Mw = 7.8) [11].

## 3. GPS Data in Ecuador

The first regional GPS research to provide direct measurements of displacement was carried out from 1988 to 1998 under the CASA project (Central and South America). They evidence that motions are due to the relative displacements of the Caribbean, Cocos, Nazca and South American plates, supporting that subduction at the western margin of North Andean Sliver is oblique at present [18,19,24]. Moreover, the CASA GPS data evidence a partitioning of continental deformation that is highly controlled by the diverging motion of two continental slivers: the Inca Sliver in northern Perú and southern Ecuador and the North Andean Sliver in Ecuador and Colombia [15]. These relative motions are linked to the sequence of great earthquakes during the last century [10,11,15,18,53,54]. The GPS studies evidence in addition the creep character of active faults in the Andes [55], accommodating deformation and decreasing their related seismicity.

GPS data in Ecuador were obtained from non-permanent stations of the RENAGE network [26] installed by the IGM (Ecuadorian Military Geographical Institute). This network has 135 sites throughout Ecuador, and measurements go back to 1994, 1996 and 1998. The Reference Frame corresponds to SIRGAS95, ITRF94, reference time 1995.4. 

**Figure 4:**
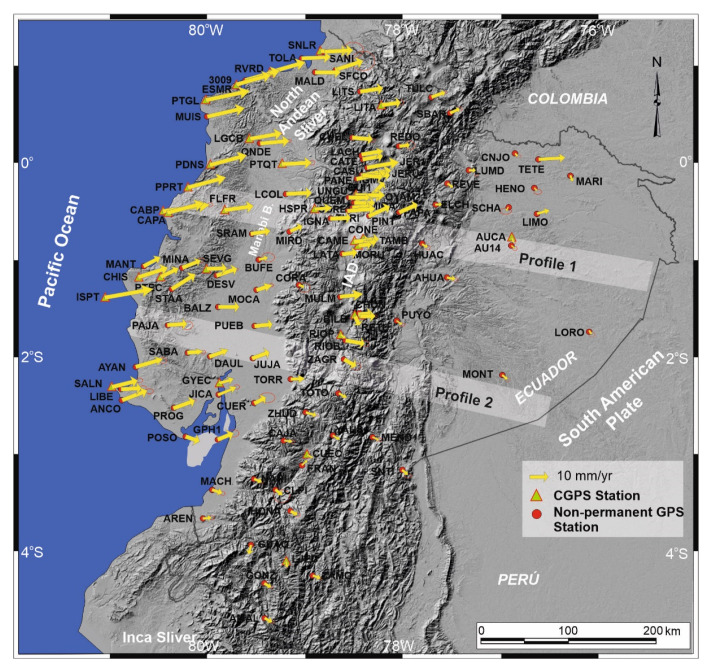
Velocity field of continental Ecuador, considering the South American stable plate reference. Error ellipses of 95% confidence. Modified from [26]. Locations of profiles 1 and 2 of Figure 5 are indicated.

The network used in this research comprises 24 CGPS stations of REGME and 87 non-permanent sites measured over 15 years, from April 1995 to January 2011 (Figure 4). Twenty-four non-permanent stations were discarded because they do not allow enough GPS measures to establish the calculation of rates. The Station Position at epoch 2011.0 and velocity were estimated with GAMIT/GLOBK software [56], expressed in the IGS08 reference frame (Table 1). The residual velocity field was computed with respect to the South American fixed reference frame (Figure 4, Table 1).

## 4. Tectonic Displacements from GPS Data

Residual velocity vectors with respect to the stable South American Plate are heterogeneous, generally with an E to ENE trend. They have greater magnitude by the coast, reaching up to 36 mm/yr, then decreasing towards the Andean region and the Oriente Basin as well as in the Inca Sliver (Figure 4). 

The Oriente Basin is characterized by low magnitude displacements, ENE to SE (AHUA, 3.9 mm/yr; LUMD, 3.5 mm/yr; SNTI, 4.3 mm; MONT, 1.8 mm/yr; PUYO, 3.5 mm/yr; AUCA, 2.3 mm/yr; LORO, 1.7 mm/yr and HENO, 2.2 mm/yr), of variable trend, that in general have an eastward component. The anomalous high TETE rate (16.6 mm/yr) is considered to be a local effect (Figure 4). 

In the Andes cordillera, most displacements have an eastward component, yet with variable trends and magnitudes. In the northern part, between ~1°30′ S and 0°45′ N, the displacement has a homogeneous ENE trend, though magnitude varies (JER1, 8.9 mm/yr; LITS, 13.4 mm/yr; TURI, 14.5 mm/yr; PAPA, 9.9 mm/yr; LITA, 12.3 mm/yr; UNGU, 12.1 mm/yr; CUEL, 12.5 mm/yr; LANCH, 10.1 mm/yr; RETU, 10.0 mm/yr; CONE, 7.9 mm/yr; LATA, 9.3 mm/yr; RIOP, 5.3 mm/yr; REVE, 4.0 mm/yr) (Figure 4). However, south of 1°30′ S, the trends change to SSE and the rates are lower (TOTO, 5.7 mm/yr; ZHUD, 6.0 mm/yr; GUAQ, 4.5 mm/yr; ZAMO, 4.5 mm/yr; CAJA, 5.1 mm/yr; GONZ, 4.0 mm/yr; CUEC, 4.5 mm/yr; HONA 3.8 mm/yr and LJEC, 4.5 mm/yr). 

In the northern coastal region the displacements are generally ENE-ward up to 2°30′ S. Rates are high along the coast and decrease towards the Manabí Basin, then increase eastward toward the margin of the cordillera Occidental (CAPA, 25.9 mm/yr; PTGL, 24.3 mm/yr; PDNS, 19.4 mm/yr; RVRD, 21.7 mm/yr; MUIS, 20.6 mm/yr; PTEC, 13.1 mm/yr; CHIS; 17.0 mm/yr; FLFR, 15.3 mm/yr; ANCO, 9.9 mm/yr; PTQT, 15.8; FLFR, 15.3 mm/yr; SNLR, 15.2 mm/yr; SFCO, 13.0 mm/yr; SALN, 13.7 mm/yr; QNDE, 15.8; PROG, 10.3 mm/yr; LCOL, 13.4 mm/yr; PAJA, 12.3 mm/yr; BALZ, 10.6 mm/yr; MINA, 10.8 mm/yr; DAUL, 11.1 mm/yr; JUJA, 10.1 mm/yr; PUEB, 10.3 mm/yr; MOCA, 10.1 mm/yr; SRAM, 13.1 mm/yr; BUFE, 7.3 mm/yr; MIRD, 9.2 mm/yr). This setting indicates shortening in the western Manabí Basin and moderate extension at its eastern margin (Figure 4). Along the southern coast, rates and trends become low and variable. 

## 5. Active Tectonic Deformations

**Figure 5:**
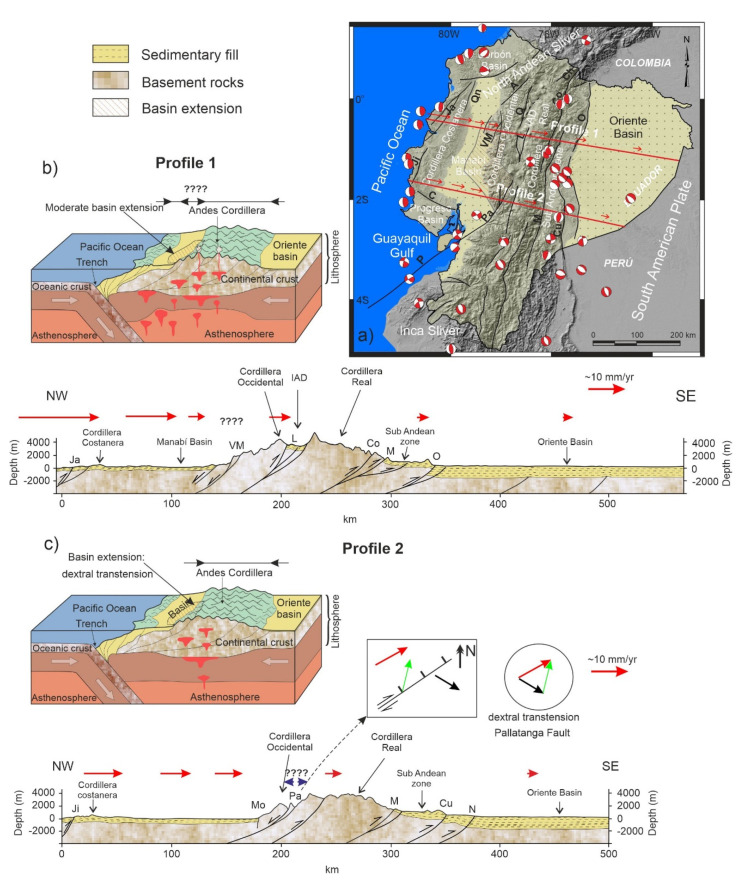
Tectonic sketch of the main active structures across the South American Plate margin in Ecuador based on the velocity field plotted in Figure 4. (**a**) Distribution profiles along the Ecuadorian Andes. (**b**) Profile 1. (**c**) Profile 2. The dextral transtensional character of the Pallatanga fault is underlined. Oriente fault, O; Napo fault, N; Cutucú fault, Cu; Méndez fault, M; Chingual fault, Ch; Cosanga fault, Co; Quito fault, Q; Latacunga fault, L; Apuela fault, A; Pallatanga fault, Pa; Puná fault, P; Valencia–Maná fault, VM; Buena Fé fault, Bn; Daule fault, D; Quinindé fault, Qn; Jama fault, Ja; Jipijapa fault, Ji; Colonche fault, C.

The active tectonic deformation evidenced by the RENAGE and REGME GPS network [26] (Figure 4) helps discern the main fault zones that are related to the crustal seismic activity in Ecuador. Most displacements have an ENE-component (Figure 4), whereas the Andes has a NNE–SSW strike supporting a regional transpressive dextral deformation along the mountain belt. In this setting, two E–W profiles roughly orthogonal to the geological structures are analyzed to observe the distribution of extensional and compressional deformations (Figure 5). 

We use GPS data to quantify the surface deformation with respect to the stable South American Plate along two WNW–ESE profiles orthogonal to the Andes Cordillera (Figure 5a). Profile 1 (Figure 5), located to the north, at roughly 1° S, is characterized by a regional ENE displacement from the coastline and Cordillera Costanera, Manabí Basin and the central Ecuadorian Andes that becomes SE toward the Oriente Basin, with variable rates (Figure 4). This profile (Figure 5b) shows eastwardly a progressive decrease of deformation, from the Cordillera Costanera towards the Manabí Basin that would indicate shortening (CAPA; 25.9 mm/yr; FLFR, 15.3 mm/yr; SRAM, 13.1 mm/yr; MIRD, 9.2 mm/yr), followed by an increase of deformation in the eastern border of Manabi Basin and across the Cordillera Occidental (MIRD, 9.2 mm/yr; IGNA, 10.6 mm/yr;) suggesting an stable or even an extensional area, and finally decreased rates along the Andes Cordillera (CAME, 9.9 mm/yr; HUAC, 5.0 mm/yr; LORO, 1.7 mm/yr) up to the Oriente Basin, related to shortening. This deformation is accommodated by several fault zones (f.z.): the compressional Jama f.z. in the Cordillera Costanera; the extensional Valencia-La Maná f.z. in the eastern Manabí Basin and western Cordillera Occidental; the compressional Latacunga f.z. in the Inter-Andean valley; the Cosanga f.z. and Sub-andean zone in the Cordillera Real followed by the Oriente fault in the boundary with the Oriente Basin. The Cordillera Costanera recent uplift was controlled by the NE–SW oriented Jama fault zone; the highest tectonic displacement rates towards the Ecuadorian margin are found here. The Cordillera Occidental is separated from the Manabí Basin by the Valencia–La Mana fault zone [57], which has very moderate activity. This westward-dipping fault has a normal component and accommodates the extension of the eastern part of the basin. The Latacunga fault zone is reverse and is located in the Inter-Andean Depression. To the west, the Cosanga fault zone accommodates shortening on the eastern edge of the Cordillera Real with reverse and minor dextral kinematics. The Oriente Basin corresponds to the stable South American Plate, separated from the Sub-Andean zone by Oriente fault.

The southern profile 2 (Figure 5c) corresponds to a section along approximately 2° S located at the southern end of the Inter-Andean Valley, where the Cordillera Occidental is very close to the Cordillera Real, and also crosses the southern end of Manabi Basin. It is characterized by an eastward decrease in deformation (PAJA, 12.2 mm/yr; DAUL, 11.1 mm/yr; JUJA, 10.1 mm/yr; TOTO, 5.7 mm/yr; MONT, 1.8 mm/yr) separated in a western sector with ENE displacements and an eastern sector with ESE displacements. Deformation is accommodated by several fault zones: the Jipijapa f.z. in Cordillera Costanera, Mo and Pallatanga Fault in Cordillera Occidental and Macas, Cutucú and Napo f.z. in the boundary between Cordillera oriental and Oriente Basin. The western sector near the coastal margin has the highest ENE displacement rates accommodated by the reverse Jipijapa fault linked to recent uplift of the relief in the Cordillera Costanera. The Mo reverse fault is located at the eastern boundary of the Manabi basin. The Pallatanga fault in the Cordillera Occidental is a main structure that determines the sharp boundary between sectors of ENE-ward displacement corresponding to the North Andean Sliver and ESE-ward displacements of the South American Plate. The GPS data (Figure 4 and Figure 5c) support the interpretation that the Pallatanga fault zone has normal and dextral kinematics in the intersection of this profile. In the eastern sector, the Macas fault accommodates shortening on the eastern edge of the Cordillera Real with reverse kinematics. Eastwards are located the Cutucu f.z. that shape the contact with the Sub-Andean zone of moderate activity. Finally, in the boundary is located the stable Oriente Basin that belongs to the South American Plate. 

## 6. Discussion

**Figure 6:**
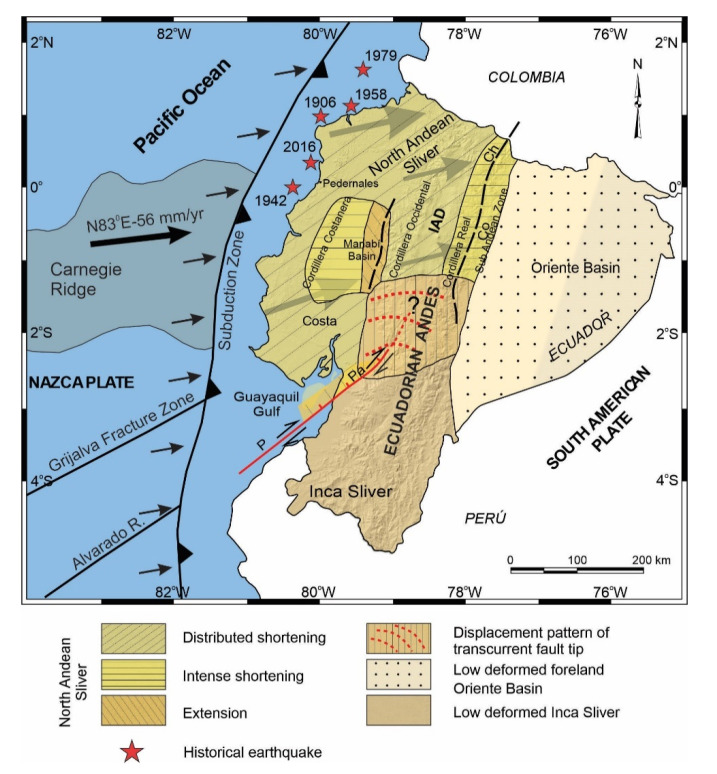
Tectonic model based on the velocity field of Ecuador. The North Andean Sliver underwent shortening and extension in the Manabí Basin. The Puná–Pallatanga transfer fault separated the North Andean the Inca Sliver. The low deformed Oriente Basin belongs to the stable South American Plate. Puná Fault, P; Pallatanga Fault, Pa; Cosanga–Chingual fault, CoCh; Inter-Andean Depression, IAD.

In view of previous results and proposals of tectonic domains and major structures (Figure 1) [2,16,17,18], a new tectonic zonation of active tectonic structures is proposed for Ecuador, divided into domains with homogeneous behavior including areas of distributed shortening, intense shortening, extension, low deformed and areas of variable behavior affected by transcurrent fault tip (Figure 6). The North Andean Sliver is affected by shortening and local extension, with the highest ENE displacement rates of the Ecuadorian margin controlled by important westward-dipping faults (Figure 4, Figure 5 and Figure 6) and intense seismic activity (Figure 3). The eastwardly progressive decrease in velocities from coastal areas (Figure 4 and Figure 5) indicates shortening and reverse faulting affecting the Cordillera Costanera, Cordilleras Occidental and Real that determines the relief uplift. Extension is associated with the normal faulting of the eastern Manabí Basin. The Inter-Andean Depression, located between the Cordillera Occidental and Real, formed as a consequence of the Andes uplifting, controlled by the Quito and Latacunga fault zones. The intense shortening in the Cordillera Real is controlled by the Cosanga–Chingual fault zone between the Sub-Andean zone, extending towards the Oriente Basin. The progressive decrease of the displacement and seismicity in the Oriente Basin would indicate a stable zone within the South American Plate. Relevant seismic activity occurs at the margin of the Sub-Andean zone, within the southern Ecuadorian Andes, with hypocenters reaching as much as 200 km in depth related to the subducted slab. 

The focal mechanisms of crustal seismicity (Figure 3) reflect the occurrence of earthquakes with an important strike–slip component, this northeastward sliding of the North Andean Sliver generates extensional strain and is responsible for the development of the Gulf of Guayaquil. The tectonic boundary between the North Andean and Inca Sliver is located at the Puná–Pallatanga fault. The Puná–Pallatanga fault registers seismicity of variable depths, including relevant activity at 40 to 120 km depths toward the Gulf of Guayaquil. Moreover, towards the northeast fault tip, seismicity reaches depths of 120 to 240 km, thereby indicating that this fault is a main lithospheric structure. Its kinematics are dextral in the Gulf of Guayaquil (Isla Puná) and become transtensional with a normal dextral component across the southern Cordillera Occidental, in the area of connection with the North–South striking faults along the Inter Andian Depression and Cordillera Real (Figure 5c). The northeastward fault tip determines an arcuate displacement field, and the progressive change of displacement vectors from NE-wards in the northern Andes, to E- and SE-wards in the southern Andes (Figure 4 and Figure 5c). Southwards, the Inca Sliver is characterized by heterogeneous low displacements. This domain probably constitutes a resistant block attached to the South American Plate that, moreover, determined the main inflection of the Pacific margin of South America. The 40 to 120 km deep earthquakes that occur in this area are related to the subducted Nazca Plate below the South American Plate.

In this setting, the NE-ward displacement of the North Andean Sliver, the high deformation rates along the Cordillera Costanera and the development of the Manabi Basin, with moderate extensional structures at its eastern boundary may be a consequence of the NE-ward pushing of the Carnegie ridge through subduction the Nazca plate and the high relative strength of the Inca Sliver attached to the South American plate (Figure 6).

The recent tectonic models [2,16] identify the importance of the transpressive Chingual–Cosanga–Pallatanga-Puná fault zone. Moreover, our results suggest that the Chingual–Cosanga segment is disconnected through a wide deformation zone from the Pallatanga–Puná segment. This last segment has a clear dextral transtensional character with slip rates close to 10 mm/yr that favors the development of the Guayaquil Gulf, separating two areas of different seismic activity: the northwestern North Andean Sliver affected by seismicity and the southeastern Inca Sliver, the most stable area (Figure 3 and Figure 6). In addition, we found that moderate deformation and seismicity also occur inside the North Andean Sliver, including areas of local extension in the Manabi Basin, and may not be considered as an undeformed block. 

## 7. Conclusions

The Ecuadorian Andes develops parallel to the South American continental margin and currently undergoes regional heterogeneous ENE–WSW shortening and local areas of extension as a result of the Nazca plate subduction and the presence of the North Andean Sliver. Altogether, active faults, earthquake focal mechanisms and GPS displacement allow the region to be divided in several domains (distributed shortening, intense shortening, extension, stable and other settings) according to active tectonic structures (Figure 6). In the North Andean Sliver, the ENE displacement of up to 36 mm/yr in the coastal areas, relative to stable South America, generally decreases eastwards indicating shortening in the Cordillera Costanera, the Cordillera Occidental and Cordillera Real, with a sharp displacement decrease with rates of down to 4 mm/yr at the eastern frontal Sub Andean Zone, in contact with the stable Oriente Basin foreland. The Manabí fore-arc basin, developed in between Cordillera Costanera to the west and Cordillera Occidental to the east, is currently affected by moderate extensional deformations at its eastern boundary. 

The highest ENE displacement and shortening rates, that affect the North Andean Sliver, in addition to the development of the Manabi fore-arc basin, are probably determined by the push of the Carnegie Ridge and the coupling of subduction. The North Andean domain is bounded to the south by the Pallatanga fault zone, the dextral transtensional character of which contributes to the Guayaquil Gulf development and determines a radial displacement pattern in the fault tip when it reaches the Andes Cordillera. In contrast, the low deformation rates in the Inca Sliver and the South Ecuadorian Andes are a consequence of the presence of rigid basement elements attached to the South American Plate craton. 

The main seismic events of Ecuador related to tectonic activity [4,6,8,10,11], with a recurrence between 16 and 37 years, are located in the subduction zone along the Pacific margin where the last major earthquake occurred (16 April 2016; Mw = 7.8; 20 km depth) in the area of Pedernales as a result of the propagation of thrust activity. Moreover, in continental areas the main active structures are located in the boundaries of the main domains: the Pallatanga fault zone, at the southern boundary of the North Andean Sliver and the Chingual–Cosanga fault zone in the eastern Andes Mountain front. 

The GNSS observations constitute the best tool to characterize the variability of the behavior of active major faults that deform oblique convergent continental margins. They contribute to determining the strain partitioning along the margin and finally to highlight areas of highest geological hazards related to tectonic deformation and seismicity. 

## Figures and Tables

**Table 1:** Absolute velocities in North and East components from GPS position time series in IGS08 frame, epoch 2011.0 and 1-σ uncertainties. Residual velocities with respect to South America fixed reference frame.

	Geodetic Coordinates (deg.)		Velocity (mm/yr)		Uncertainty (mm/yr)		Residual Velocity (mm/yr)	
Station	Lat. (N)	Long (E)	VEast	VNorth	sVEast	sVNorth	VrEast	VrNorth
3009	0.9890	−79.6265	14.6	15.3	0.5	0.3	19.5	6.2
AHUA	−1.0610	−77.5500	−0.8	8.9	0.9	0.4	3.8	−0.6
AMAL	−4.5812	−79.4266	0.1	7.1	0.8	0.4	4.2	−2.1
ANCO	−2.3296	−80.8947	4.6	13.0	1.0	0.5	9.1	4.1
AREN	−3.5591	−80.0685	1.5	7.7	1.5	0.9	5.8	−1.4
AU14	−0.7301	−76.8867	−2.4	9.0	1.2	0.6	2.3	−0.5
AUCA	−0.6408	−76.8827	−3.4	7.5	1.2	0.9	1.2	−2.0
AYAN	−1.9885	−80.7569	8.6	12.4	1.8	0.9	13.1	3.4
BALZ	−1.3675	−79.9086	6.0	9.5	3.5	1.8	10.6	0.4
BILB	−1.4466	−78.5016	−4.4	6.9	1.1	0.7	0.1	−2.3
BUFE	−0.8771	−79.4884	2.3	11.6	4.1	1.8	6.9	2.5
CABP	−0.3865	−80.4287	12.9	12.3	0.9	1.0	17.6	3.3
CAJA	−2.7531	−79.2370	0.5	7.8	0.9	0.5	4.9	−1.4
CAME	−0.6797	−78.5063	5.1	10.7	1.0	0.4	9.8	1.4
CAPA	−0.3689	−80.4732	20.8	12.9	1.4	0.4	25.6	3.9
CASI	−0.0366	−78.4802	6.9	11.4	2.0	1.0	11.7	2.1
CATE	0.0002	−78.4284	5.7	9.3	1.6	0.7	10.5	0.0
CHIS	−1.0531	−80.7284	11.2	15.2	0.6	0.4	15.8	6.2
CHON	−1.4331	−78.4632	1.1	9.7	0.9	0.4	5.6	0.5
CLPI	−3.2549	−79.3200	0.0	5.9	3.5	1.9	4.3	−3.2
CNJO	0.2381	−76.8447	−2.8	9.5	1.5	0.7	2.0	0.0
CONE	−0.6600	−78.4137	3.2	10.3	0.6	0.4	7.9	1.0
CORA	−1.1381	−79.0792	2.3	10.8	1.6	0.8	6.9	1.6
CORE	−0.3278	−78.5241	5.5	10.9	1.4	0.6	10.3	1.6
CUEC	−2.8833	−79.0025	−0.3	7.3	0.8	0.6	4.1	−2.0
CUEL	0.3895	−78.5328	7.7	8.8	1.4	0.7	12.5	−0.5
CUER	−2.3587	−79.5328	1.0	12.1	1.3	0.7	5.4	3.0
DAUL	−1.8766	−79.9955	6.2	12.1	4.8	2.0	10.6	3.0
DESV	−1.0405	−79.9240	6.4	12.6	0.7	0.4	11.0	3.5
ELCH	−0.3345	−77.8064	−0.8	10.2	0.7	0.4	3.9	0.8
FLFR	−0.3574	−79.8427	10.5	11.0	0.9	0.8	15.2	1.9
FRAN	−3.0078	−79.0431	−3.6	11.8	2.7	1.6	0.7	2.6
GONZ	−4.2264	−79.4307	−0.7	7.1	0.7	0.4	3.5	−2.1
GPH1	−2.7372	−79.9112	1.8	10.9	2.0	1.1	6.2	1.9
GUAQ	−3.8304	−79.5748	−4.9	4.7	3.4	1.8	−0.6	−4.4
GYEC	−2.1494	−79.8919	4.4	13.8	0.8	0.7	8.9	4.7
HENO	−0.1319	−76.6490	−2.6	9.0	1.7	0.7	2.1	−0.5
HONA	−3.4765	−79.1599	−0.8	7.6	0.6	0.4	3.5	−1.6
HUAC	−0.7073	−77.8049	0.2	8.2	0.9	0.5	4.9	−1.2
IGMV	−0.2151	−78.4936	7.0	9.3	1.5	0.7	11.7	0.0
IGNA	−0.4509	−78.7519	5.6	11.8	0.7	0.4	10.3	2.5
ISPT	−1.2621	−81.0736	31.1	14.8	1.0	0.5	35.6	5.9
JER1	−0.0054	−78.3580	3.9	11.6	1.9	0.9	8.6	2.2
JERU	−0.0056	−78.3580	5.0	11.7	0.6	0.3	9.8	2.4
JICA	−2.2718	−79.9039	4.0	12.8	1.5	0.8	8.4	3.7
JUJA	−1.8943	−79.5539	5.0	12.6	1.3	0.6	9.5	3.4
LACH	0.1610	−78.4200	5.0	11.9	5.0	2.4	9.7	2.6
LATA	−0.8139	−78.6265	4.5	10.8	0.5	0.3	9.2	1.5
LCOL	−0.2499	−79.2037	8.4	11.6	0.7	0.4	13.1	2.5
LGCB	0.3821	−79.5753	12.1	12.5	0.8	0.5	16.9	3.3
LIBE	−2.2191	−80.9051	7.6	10.4	1.6	1.0	12.0	1.5
LITA	0.7263	−78.2187	7.2	12.0	2.1	1.4	12.0	2.7
LITS	0.8702	−78.4480	8.3	11.7	0.6	0.3	13.2	2.4
LJEC	−3.9883	−79.1985	−0.7	6.2	2.9	0.8	3.5	−2.9
LORO	−1.6132	−75.9869	−5.1	11.3	1.7	0.8	−0.5	1.6
LUMD	0.0083	−77.3222	−1.3	9.6	1.0	0.4	3.5	0.2
MACH	−3.2565	−79.9685	1.8	9.3	1.8	1.0	6.1	0.2
MALD	1.0701	−78.9089	6.7	9.5	7.9	4.0	11.6	0.3
MANT	−0.9366	−80.6712	11.8	12.6	3.0	1.6	16.5	3.6
MARI	−0.0496	−76.2951	−2.7	8.6	6.2	2.8	2.0	−1.0
MEND	−2.7175	−78.3196	0.1	7.6	1.0	0.5	4.5	−1.7
MINA	−0.9632	−80.2788	5.6	12.5	1.2	0.6	10.3	3.5
MIRA	−0.2704	−78.5089	5.4	10.1	1.4	0.7	10.1	0.8
MIRD	−0.5827	−79.1640	3.0	14.3	2.2	1.1	7.7	5.1
MOCA	−1.1871	−79.5091	5.1	11.9	0.9	0.5	9.7	2.8
MONT	−2.0673	−76.9808	−2.7	9.6	1.5	0.7	1.8	0.1
MORU	−0.7253	−78.4591	4.2	11.1	0.8	0.5	8.8	1.8
MUIS	0.6045	−80.0238	15.2	13.9	0.5	0.3	20.0	4.9
NARI	−3.1413	−79.5365	−1.0	7.9	1.0	0.5	3.3	−1.2
OYAM	−0.2034	−78.3285	0.5	19.6	10.0	4.8	5.3	10.3
PAJA	−1.5541	−80.4283	7.3	12.1	2.3	1.2	11.9	3.1
PANE	−0.2291	−78.5183	2.2	13.9	12.0	6.9	6.9	4.7
PAPA	−0.3809	−78.1405	4.1	13.7	0.6	0.3	8.8	4.4
PDNS	0.1114	−79.9910	13.9	14.2	0.9	0.7	18.7	5.2
PINT	−0.4201	−78.3556	5.9	14.2	4.9	2.2	10.6	4.8
POSO	−2.7102	−80.2434	3.3	8.2	4.6	2.4	7.6	−0.8
PPRT	−0.1253	−80.2165	15.4	15.7	0.8	0.5	20.1	6.7
PROG	−2.4108	−80.3654	5.4	11.9	0.7	0.4	9.8	2.9
PTEC	−1.0580	−80.4746	7.8	13.1	3.1	2.0	12.4	4.1
PTGL	0.7815	−80.0304	18.9	14.1	1.0	0.6	23.8	5.0
PTQT	0.1230	−79.2520	11.0	9.6	2.3	1.5	15.8	0.4
PUEB	−1.5589	−79.5307	5.7	10.2	1.0	0.5	10.2	1.1
PUYO	−1.5048	−78.0640	−1.5	7.7	1.0	0.5	3.1	−1.6
QNDE	0.3275	−79.4755	10.9	11.1	0.7	0.4	15.7	2.0
QUEM	−0.2371	−78.4973	8.1	11.4	0.8	0.4	12.9	2.1
QUI1	−0.2152	−78.4936	7.2	10.8	0.3	0.2	12.0	1.6
REDO	0.3044	−78.0462	1.3	10.5	2.6	1.2	6.1	1.1
RETU	−1.4518	−78.4424	5.4	9.6	2.2	0.8	10.0	0.3
REVE	−0.0473	−77.5268	−1.3	7.3	0.7	0.4	3.4	−2.1
RIOB	−1.7007	−78.5912	3.1	6.2	3.1	1.4	7.6	−3.1
RIOP	−1.6506	−78.6511	−0.6	5.7	0.5	0.2	3.9	−3.6
RVRD	1.0676	−79.3851	16.2	14.3	1.0	0.5	21.1	5.1
SABA	−1.8409	−80.2230	4.1	10.6	0.9	0.5	8.6	1.5
SALN	−2.1862	−80.9908	8.8	12.2	1.2	0.8	13.3	3.3
SBAR	0.6463	−77.5248	0.7	12.5	0.9	0.4	5.6	3.1
SCHA	−0.3264	−76.9125	−5.5	9.1	3.1	1.3	−0.8	−0.4
SFCO	1.0915	−78.7012	7.4	13.6	5.6	2.6	12.3	4.3
SNLR	1.2925	−78.8470	10.2	8.8	1.5	1.3	15.2	−0.4
SNTI	−3.0495	−78.0102	−0.8	6.9	1.2	0.5	3.5	−2.4
SRAM	−0.6100	−79.5607	8.2	11.2	3.9	1.9	12.9	2.1
STAA	−1.1827	−80.3872	4.0	12.1	0.8	0.4	8.6	3.1
TAMB	−0.6872	−78.4011	3.2	10.4	0.8	0.3	7.9	1.1
TETE	0.1769	−76.5329	11.8	10.3	6.6	1.8	16.6	0.7
TOLA	1.2103	−79.0454	11.4	10.8	0.7	0.4	16.3	1.6
TOTO	−2.2569	−78.6728	0.7	6.8	0.6	0.3	5.1	−2.5
TULC	0.8120	−77.7053	2.8	12.2	0.8	0.4	7.7	2.8
TURI	−0.3679	−78.5764	9.8	10.4	4.1	1.9	14.5	1.1
UNGU	−0.2374	−78.5572	7.3	10.4	1.0	0.5	12.0	1.1
ZAGR	−1.9075	−78.6106	−0.6	7.5	4.6	1.9	3.9	−1.7
ZAMO	−4.0548	−78.9320	−0.1	7.5	0.5	0.3	4.1	−1.7
ZHUD	−2.4613	−79.0059	1.3	7.4	0.7	0.4	5.7	−1.8

## Data Availability

The data are included in the Table 1 of this contribution.
